# ViBiBa: Virtual BioBanking for the DETECT multicenter trial program - decentralized storage and processing

**DOI:** 10.1016/j.tranon.2021.101132

**Published:** 2021-05-27

**Authors:** H. Asperger, J.-P. Cieslik, B. Alberter, C. Köstler, B. Polzer, V. Müller, K. Pantel, S. Riethdorf, A. Koch, A. Hartkopf, L. Wiesmüller, W. Janni, F. Schochter, A. Franken, D. Niederacher, T. Fehm, H. Neubauer

**Affiliations:** a Department of Obstetrics and Gynecology, University Hospital Duesseldorf, Moorenstraße 5, Düsseldorf, Germany; b Division of Personalized Tumor Therapy, Fraunhofer ITEM, Germany, Am Biopark 9, Regensburg, Germany; c Department of Obstetrics and Gynecology, University Hospital Hamburg-Eppendorf, Germany, Martinistraße 52, Hamburg, Germany; d Department of Tumor Biology, University Hospital Hamburg-Eppendorf, Germany, Martinistraße 52, Hamburg, Germany; e Department of Obstetrics and Gynecology, University of Tübingen, Germany, Calwerstraße 7, Tübingen, Germany; f Department of Obstetrics and Gynecology, University Hospital Ulm, Germany, Albert-Einstein-Allee 23, Ulm, Germany

**Keywords:** ViBiBa, Virtual biobanking, Liquid biopsy, CTC

## Abstract

**Background:**

Liquid Biopsy (LB) in the form of e.g., circulating tumor cells (CTCs) is a promising non-invasive approach to support current therapeutic cancer management. However, the proof of clinical utility of CTCs in informing therapeutic decision-making for e.g., breast cancer in clinical trials and associated translational research projects is facing the issues of low CTC positivity rates and low CTC numbers – even in the metastasized situation. To compensate for this dilemma, clinical CTC trials are designed as large multicenter endeavors with decentralized sample collection, processing and storage of products, making data management highly important to enable high-quality translational CTC research.

**Aim:**

In the DETECT clinical CTC trials we aimed at developing a custom-made, browser-based virtual database to harmonize and organize both decentralized processing and storage of LB specimens and to enable the collection of clinically meaningful LB sample.

**Methods:**

ViBiBa processes data from various sources, harmonizes the data and creates an easily searchable multilayered database.

**Results:**

An open-source virtual bio-banking web-application termed ViBiBa was created, which automatically processes data from multiple non-standardized sources. These data are automatically checked and merged into one centralized databank and are providing the opportunity to extract clinically relevant patient cohorts and CTC sample collections.

**Summary:**

ViBiBa, which is a highly flexible tool that allows for decentralized sample storage of liquid biopsy specimens, facilitates a solution which promotes collaboration in a user-friendly, federalist and highly structured way. The source code is available under the MIT license from https://vibiba.com or https://github.com/asperciesl/ViBiBa

## Highlights

• ViBiBa is an open-source sample banking tool.
• ViBiBa was purpose built for liquid biopsy specimen.
• ViBiBa allows for decentralized storage while promoting collaboration.
• ViBiBa's plugin support requires no change in existing data structures.
• ViBiBa empowers translational research projects and cohort formation.

## Introduction

Cancer is a leading cause of death worldwide and early diagnosis and precise therapy are an enormous challenge. With 24.2% of new cases breast cancer (BC) is the most frequent cancer type in women worldwide and the detection of early disease and curable stages remains difficult [1]. During evolution and progression of BC extensive genetic and molecular heterogeneity occurs leading to variability within the primary tumor mass (spatial heterogeneity) and between the primary tumor (pTu) and its metastatic spread during cancer development or following treatment (temporal heterogeneity). Conventional diagnostic tools, e.g., imaging methods, biopsies and traditional serum biomarkers do not mirror the dynamic changes and heterogenous character of the genomic and molecular tumor landscape [2,3]. In this context Liquid Biopsy (LB) — in the form of circulating tumor cells (CTCs) and circulating tumor DNA (ctDNA) — is gaining increasing value to support current therapeutic cancer management. CTCs are deemed to be evading cancer cells that have been shed or actively invaded from the primary tumor or a metastasis into the blood circulation or lymph system. While the majority of CTCs is eliminated through the immune system, escaping CTCs are able to finally extravasate to found (further) metastases [4,5]. Therefore, they are considered — at least in part — to be metastatic progenitors [6]. ctDNA is released from apoptotic or necrotic tumor cells and is normally measured in plasma or serum [7]. Recent studies revealed the strong clinical value of both CTCs and ctDNA in BC [7], [8], [9], [10], [11], [12], [13], [14], [15]. With increasing evidence for the efficacy of a CTC or ctDNA guided therapy [16,17], there is a growing need to analyze and understand the CTCs’ biology. Despite the promising possibilities of LB its clinical application remains limited due to missing clinical validity and utility. In order to transfer LB in the clinical routine, standard operating procedures (SOPs) for LB workflows related to the collection and extraction of reference material, sample preparation, logistics and analysis need to be implemented [18]. Low positivity rates in combination with the small amount of the target material make it necessary to create sufficient cohorts [19]. These challenges are approached by associating translational research projects to different multicenter clinical trials. Since both disciplines may aim at different parameters, this endeavor harbors different issues including decentralized gain and processing of samples, a need for detailed communication and the central management of patient and sample data albeit decentralized banking. Here, we present the development of ViBiBa as an easy way of sharing data and requesting parts of samples via an intuitive and standardized workflow.

## Materials and methods

### User interface

**Fig. 1:**
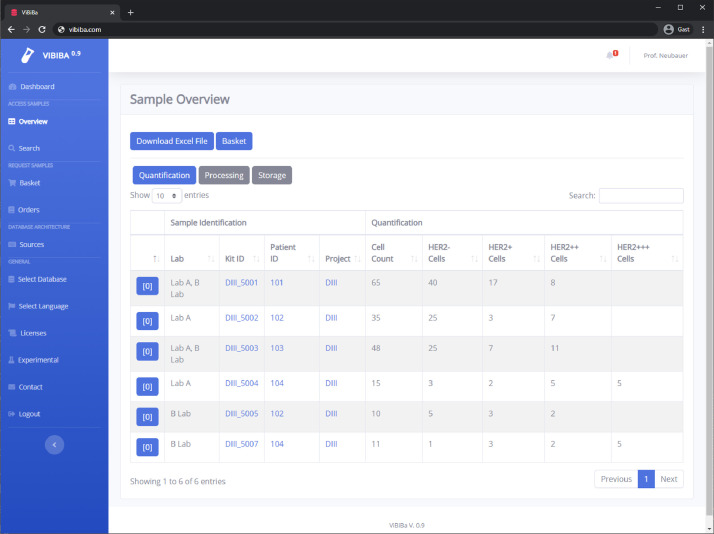
Screenshot of ViBiBa with fictive sample data.

The user interface is kept simple and multilingual. The core application uses the bootstrap framework from twitter (under MIT license) together with multiple plugins and template boilerplates, which are also mainly under the MIT license. On the left side, the user can access a retractable menu, while the right side is used to display the main content (Fig. 1) The menu is divided into "Dashboard", "Access Samples", "Request Samples", "Database Architecture", "General" subsections. The user can change the currently viewed database and the display language in the menu.

### Basic structure

**Fig. 2:**
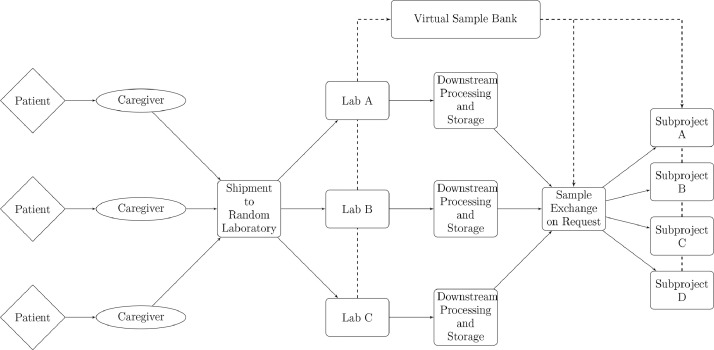
Logistics in the DETECT trial program Schematic of the basic (virtual) logistics between the participating DETECTlaboratories.

**Fig. 3:**
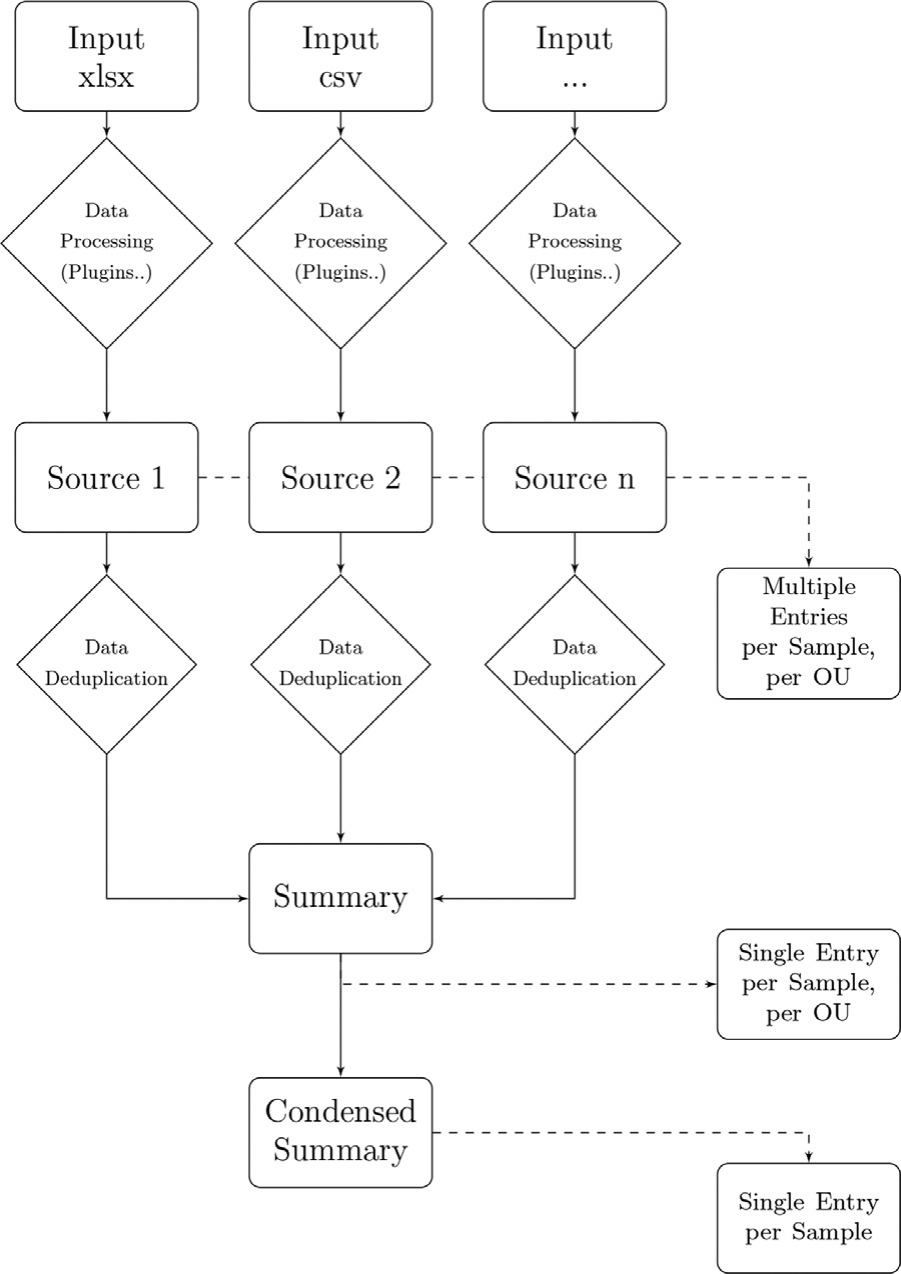
Architecture of ViBiBa Schematic of the internal data structure and processing.

One of the main goals of ViBiBa is to allow for samples to be shipped to different laboratories, while maintaining the ability for joined translational research projects (Fig. 2). To achieve this, the participating laboratories must upload data in some form, which is then processed by ViBiBa to allow for sample exchange and cohort management. ViBiBa's back end is written in PHP and MySQL. Users can access the database via a browser interface. As described in Fig. 1 ViBiBa consists of three layers of data: multiple sources, one summary and a condensed summary. The farther the data is processed, the denser the data gets. While the data directly supplied by the laboratories, can be stored with arbitrary column definitions and formats, the data is then processed to fit into a strictly defined list of fields (Table 1f) and (Fig. 3).

**Table 1:** Exemplary database fields

Identification	CellSearch	Kit Shipment	Single Cell Isolation	Iso. Cells (Count)	Storage
Lab	Entry Date	CS: Shipment	Date of Isolation	CD45-/EpCAM+	Serum Aliquots (ml)
Kit ID	Determination Done	CS: Date	CellCelector	CD45-/EpCAM-	Serum bank (µl)
Patient ID	Reason if no Determ.	CS: Destination	DEPArray	CD45+/EpCAM-	Box Position
Study Arm	Determ. Date	CS2: Shipment	FACS	No Cell Control	Comment
[Origin]	Time till Determ.	CS2: Date	Manual Isolation	Other (single cells)	
	Blood Volume (ml)	CS2: Destination	Isolated Cell Count		
	Cell Count	EDTA: Shipment	Buffer Water		
	HER2-negativ cells	EDTA: Date	Buffer PBS		
	HER2+ Cells +	EDTA: Destination	Deposition Format		
	HER2+ Cells ++	Cartr.: Shipment	Count Deposition		
	HER2+ Cells +++	Cartr.: Date			
	Tumor cell count	Cartr.: Destination			

Excerpt of the database fields currently used in the DETECT production version of ViBiBa. The fields are user defined and must match a column type from Table 2.

CS = Cellsave Preservative Tubes; Cartr. = Cartridge; Iso. = Isolated

### Sources and inputs

Every organizational unit can create an unlimited number of sources, which are the foundation of the internal data processing. Changes to the data must be done directly on the sources. Build in plugin support allows for custom data transformation and filtering. The interface can accept multi-worksheet .xlsx files, which are either mapped to multiple tables (which are subsequently united via a plugin) or read as a single continuous table. Users can specify to skip the first n rows of the table in case the table contains additional information such as extensive column descriptions. The first row that is read, must specify the internal column names, this allows for future changes in the .xlsx files. The interface intentionally allows the user to display every single source (even outside of their organizational unit) to make the database more transparent.

### Summary table

The summary table contains an organized overview of all available samples. Samples can have multiple entries (one per organizational unit). As every source can have an unlimited number of entries per sample, ViBiBa needs to decide how to process conflicting data. Firstly, the sources are individually deduplicated. When multiple information for a specific sample in a specific organizational unit is present, the most recent row overwrites the others, and a warning is shown in the processing log. Afterwards, the different sources are merged, and the process is repeated. Every source has an assigned priority. If multiple sources offer conflicting data (per sample per organizational unit) the higher priority source overwrites the others.

### Condensed summary table

The most human readable table of ViBiBa is the ’condensed summary’ table. Here only one entry per sample and organizational unit exists. To achieve this the data from the summary table must be condensed. The process is specific to the data type of the column (Table 2). This allows the user to get an overview over all samples in all study branches.

**Table 2:** Column data types

Type	Behavior on Condensation	Example			
		OU 1	OU 2	OU 3	Condensed
*Numeric*	Addition	2	5	1	8
*Numeric [multidimensional]*	Addition of the matching dimensions, afterwards concatenated to string	2/7/1	0/0/3	1/1/5	3/8/9
*Boolean*	"TRUE" if at least one entry states "TRUE"	FALSE	TRUE	FALSE	TRUE
*String/Text*	Concatenate strings [except identical entries]	"Text A"	"Text B"	"Text A"	"Text A, Text B"

Every column is assigned to a special type. Depending on the type the fields are treated differently while being processed.

## Results

### Definition of requirements

At first, we conducted an extensive literature and online search for a database application that fulfilled the needs for our trial group. We established the following criteria that the database should meet:

• Organize samples from different trial arms processed in different locations.
• Merge information gracefully concerning the same sample.
• Keep underlying information/data separated and restrict update rights to only one organizational unit per data source.
• Plugin support for data pre-processing of highly individual data sources that may contain human errors.
• Ability to self-host the (ideally open source) application for optimal control over the trial data.

### Design of ViBiBa functionality

**Fig. 4:**
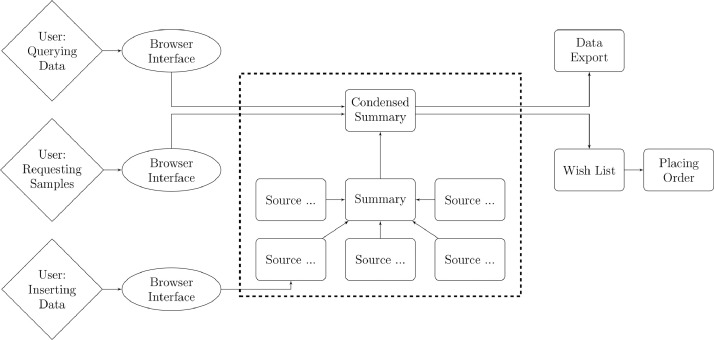
Workflow of selected processes The data flow when retrieving and manipulating information in ViBiBa.

One main concern was to allow for easy access through a browser interface. The basic functionality of ViBiBa can be broken down into "Exploring Data", "Searching Samples", "Requesting Samples" and "Inserting Data" (Fig. 4). During everyday usage, the user is mainly shown data from the "condensed summary" table. But if more detailed information about a sample is required the data flow switches seamlessly to the "summary" table, where the information is divided by the contribution of the individual organizational units.

### Exploring data

While browsing the available data, the platform will fetch the basic information about the samples from the summary table. On request the user can view detailed information about a specific sample. This is useful when determining which organizational units contributed data and store physical parts of the sample. As the database also stores information about performed sample processing and analysis, the user can learn about potentially available data from the different organizational units.

### Searching samples

The platform allows the user to search the database via a custom search form. Based on the type of field the user can set a target value and select an operator (<, >, =). Multiple filters are combined with an AND gate. Afterwards, the condensed summary table is queried, and the results are presented in the browser with the ability to download the data in tabular form. This functionality is especially powerful in combination with the "request sample" function, which is described below, as the user can add all or part of the matching samples to the wish list.

### Requesting physical samples

One drawback of decentralized sample storage is the difficulty in ordering specific samples. ViBiBa implements an automated system in sample requesting. While using the web interface to search for specific samples the user can add a sample to the ’wish list’ by clicking on a check box. This process is comparable to an online shopping experience. After selecting the desired samples, one can then specify the request and select a priority for it. When the order is placed the administrators of the trial program are notified in case, they want to place a veto against the order to protect certain samples for future research projects. If the order gets accepted every organizational unit which stores part of the samples involved in the request is notified.

### Automatic flagging

One of the main advantages of a central data service in a decentralized setting, is the creation of cohorts. While the DETECT consortium, activity encourages its members to use all samples for translational research, some samples can generate additional value when combined into a cohort. To avoid that a valuable sample will be used and eventually destroyed in the process, we allow for flagging of samples. The user will see a flag next to the entry in the databank to inform about a potential designation of the sample for a cohort.

### Harmonization

While some measurements like sample volume have standardized data formats, many other parameters evaluated in our trial program are determined and recorded by the specific standards of the participating member. Downstream working with LB samples requires highly specialized equipment which may vary between centers and centers have mostly independent workflows with different methods of data storage.

### Administration

ViBiBa uses a permission system on a per-organizational unit basis. This allows to limit the user to only upload data from its own organizational unit while giving "read only" access to the whole dataset. Additionally, ViBiBa supports multiple databases so one can create a separate instance e.g., for a second trial program or for internal use.

### Data protection

To meet the requirements of data security and protection in accordance with the General Data Protection Regulation (GDPR) ViBiBa separates any patient data from the sample specific data. For the DETECT trial, no clinical patient information is deposited in the sample bank. Extension of the databank to house patient data is possible in the future but comes with deeper implications for data security, which need to be addressed first. ViBiBa is built to be run in docker containers, to allow for a more secure environment and easier development.

## Discussion

The analysis of LB derived CTCs and ctDNA is an exciting approach to complement classical tumor diagnosis for therapy selection. However, especially the use of CTCs for therapy prediction is facing the dilemma that our knowledge on the CTCs’ biology and evolution during certain treatments, which is indispensable to uncover their clinical value, is limited or not available. As an example, the German DETECT-trials [20] investigate peripheral blood from metastatic breast cancer (MBC) patients which are collected in a 10 ml tube containing a fixative. These tubes are then shipped to four expert laboratories for further analysis. Since the key challenges to CTC trials are the low positivity rates and the small amounts of the target material, DETECT is recruiting patients from more than 100 clinical centers throughout Germany [21]. After processing the blood in the CellSearch system, the products are shipped between the laboratories for various reasons one being the highly specific equipment that is required to further process and analyze single cells and that is not available at every site [22,23]. This decentralized approach comes with an inherent problem of keeping track of available data.

A further complicating issue is that different CTC laboratories run their local repositories with more or less home-brew documentation systems. Further, for the sake to avoid any bias in the clinical part of the study, potential follow-up samples from the same patient are randomly distributed between the laboratories. On top of these logistic aspects different protocols applied for detection, isolation, banking, and downstream analytical methods contribute to highly heterogeneous sample qualities making multiparametric and longitudinal investigation of CTCs with highly sensitive methods almost impossible putting this important analyte at risk of being dropped by oncologists. Even in situations outside of multicenter trials there is a growing need for virtual biobanking solutions [24]. Therefore, a data management tool covering all these aspects is highly needed.

There are different database solutions on the market to manage tissue samples or similar specimen. The BioSamples database provided by EMBL-EBI is a web portal that allows the submitter to store information about bio samples and their relationship to external databases like ArrayExpress [25]. The data is then publicly available. Data access and modification through an API is available. First published in 2006 the European Human Frozen Tumor Tissue Bank (TuBaFrost) established a consortium with rules and infrastructure for a virtual sample bank [26]. TuBaFrost was centered around the idea of retained custodianship, participating centers would only share meta data about stored samples and whether they are available for research or currently in use or out of stock. The project was discontinued partly after the data upload procedure proved to be too elaborate and participating centers showed little interest in uploading too much information [27]. Building on the advances of TuBaFrost a new database for the EuroBoNeT (European bone tumors network) was created [27]. Initially created as a direct copy of TuBaFrost it adapted new technologies allowing for virtual storage of more tissue types.

None of these databases is specifically designed for the needs of CTC or LB research associated to clinical trials. But in order to guarantee translational research on the highest quality different specific information on pre-analytic and analytic procedures are needed: there are e.g., almost a myriad of blood preservation tubes or antibody cocktails, cell labeling protocols, isolation methods for CTCs on the market influencing the compatibility and quality of a specific downstream application [28]. There are also different methods of genome amplification methods available, which influence the outcome of DNA analysis. Already this small selection of variables is implying the risk of becoming trapped in the lows of testing parameters when samples are not documented correctly. With ViBiBa we present an open-source sample bank solution purposely built for the management for the requirements of LB research.

• Automated data summary while allowing to trace back individual entries to their source.
• Individual data sources with permission managed browser upload forms and scripted service routines that sanitize the user input via plugins.
• An ordering system to allow for easy requests of samples between the participating organizations.
• Open Source and the possibility to host the application on our own servers for better data protection.

## Conclusion and outlook

We have created the working sample bank ViBiBa for our trial program and published the source code under the MIT license which has been implemented by the DETECT Consortium to improve translational LB research. We plan to extend the analytic possibilities of ViBiBa to allow for hypothesis generation inside the web interface.

## Declaration of Competing Interest

All authors have read the journal's policy on disclosure of potential conflicts of interest. We declare no conflicts of interest.
